# A Predictive Mimicker of Fracture Behavior in Fiber Reinforced Concrete Using Machine Learning

**DOI:** 10.3390/ma14247669

**Published:** 2021-12-12

**Authors:** Sikandar Ali Khokhar, Touqeer Ahmed, Rao Arsalan Khushnood, Syed Muhammad Ali, Shahnawaz

**Affiliations:** Nust Institute of Civil Engineering (NICE), School of Civil and Environmental Engineering (SCEE), National University of Sciences and Technology (NUST), Sector H-12, Islamabad 44000, Pakistan; skhokhar.bece18nice@student.nust.edu.pk (S.A.K.); tahmed.bece18nice@student.nust.edu.pk (T.A.); sali.bece18nice@student.nust.edu.pk (S.M.A.); shahnawaz.bece17nice@student.nust.edu.pk (S.)

**Keywords:** FRC, machine learning, predictive model, efficient mimicker, post-peak response, fracture behavior, mechanical properties, ductility, strain hardening

## Abstract

Due to the exceptional qualities of fiber reinforced concrete, its application is expanding day by day. However, its mixed design is mainly based on extensive experimentations. This study aims to construct a machine learning model capable of predicting the fracture behavior of all conceivable fiber reinforced concrete subclasses, especially strain hardening engineered cementitious composites. This study evaluates 15x input parameters that include the ingredients of the mixed design and the fiber properties. As a result, it predicts, for the first time, the post-peak fracture behavior of fiber-reinforced concrete matrices. Five machine learning models are developed, and their outputs are compared. These include artificial neural networks, the support vector machine, the classification and regression tree, the Gaussian process of regression, and the extreme gradient boosting tree. Due to the small size of the available dataset, this article employs a unique technique called the generative adversarial network to build a virtual data set to augment the data and improve accuracy. The results indicate that the extreme gradient boosting tree model has the lowest error and, therefore, the best mimicker in predicting fiber reinforced concrete properties. This article is anticipated to provide a considerable improvement in the recipe design of effective fiber reinforced concrete formulations.

## 1. Introduction

Due to brittle behavior, concrete absorbs significantly less energy as it shows an abrupt fracture in tension. Ductile materials can be coupled with concrete to improve tensile and energy absorption properties. Reinforced Cement Concrete (RCC) uses rebar to get better ductility and tensile strength. However, due to the larger diameter of the rebar, the cracks that rebar bridges are relatively larger, leading to durability issues [1]. The use of fibers has been increasing due to their enhanced mechanical and fracture properties. Fibers bridge the cracks at a micro-scale that controls crack width, improves crack resistance, and ensures better ductility.

Concrete reinforced with fibers can give either strain-softening or strain-hardening behavior. Mix that gives strain-hardening behavior is classified as Engineered Cementitious Composites (ECC). Earlier, it was thought that strain-hardening is achieved by only increasing the volume of fibers, but later it was found that fibers content is not the only controlling parameter. It is also the function of parameters such as:

- Fiber Properties: Mechanical properties, aspect ratio, and volume fraction;
- Matrix properties: Initial flaw size distribution and its mechanical properties;
- Fiber-matrix interfacial properties: Chemical and frictional bond.

These properties can be adjusted to achieve strain hardening behavior while keeping practical fibers content [2]. To effectively use the combination of all parameters, researchers have proposed different criteria and theoretical models for critical volume fraction for any given set of ECC constituents [2,3,4,5,6,7,8]. ECC possesses high ductility due to strain hardening characteristics and has the ability of self-healing and crack width control [9]. Due to its excellent properties, it has increased the load-carrying capacity of many structures, especially structures subjected to earthquake and fatigue loading [10,11].

It is very difficult to predict fiber reinforced concrete (FRC) fracture behavior based on its constituents, i.e., whether the concrete will have strain-hardening or undergo strain-softening. The design procedure for achieving strain-hardening of concrete is based on extensive experimentation. Moreover, the models developed by researchers for finding the critical fiber volume are based on certain factors that need to be found by experiments making this work quite lengthy, time-consuming, and uneconomical [12]. There are no defined guidelines for preparing a mixed design of strain hardening FRC. Consequently, there ought to be a tool to help prepare a mix with higher possibilities of undergoing strain-hardening and predicting its properties.

The development of machine learning (ML) methods has shown promising results in predicting materials properties [12,13,14]. It can consider complex datasets having multiple inputs and output variables and predict results with high accuracies [15,16]. These methods have already been applied to predict compressive strength, tensile strength, and strain capacity of high-performance fiber-reinforced cementitious composites (HPFRCC) [12] using 387 samples. The study by Guo et al. includes the data of HPFRCCs samples with 14 input and 3 output variables that owe the strain-hardening behavior. However, the model has a limitation on applicability only for the HPFRCC samples having strain hardening tendency. Therefore, value addition is required by developing a model with a widened scope of practice over conventional high-performance FRC mixes after requisite training and validation sets for the two streams.

This paper proposes innovation in two areas; the first is related to the functionality and comprehensiveness of the model, which is not just limited to strain-hardening FRCs, and the second is related to the model’s accuracy. Firstly, for the first time, data of FRC, ECC, and HPFRCC are collected together. A new output parameter is introduced, which belonged to the failure classification, i.e., fracture behavior. The reason to introduce this variable is to make the model familiar with the basics of post cracking behavior, so it can predict whether the resulting mix would result in strain hardening composite or not. The second is related to performance; the performance parameters achieved are relatively better than the previously made models [12]. There are 438 data samples are cumulatively used to develop the comprehensive model for FRC containing 194 samples [17,18,19,20,21,22,23,24,25,26,27,28,29,30,31,32,33,34,35], 231 ECC, and 13 HPFRC [36,37,38,39,40,41,42,43,44,45,46,47,48,49,50,51,52,53,54,55,56,57,58,59,60,61,62,63,64,65,66,67,68,69,70,71,72,73,74,75,76,77,78,79,80,81,82,83,84,85,86,87,88,89,90,91,92,93,94,95,96,97,98] extracted from published literature. Five ML models were developed. These include artificial neural network (ANN), the support vector machine (SVM), the classification and regression tree (CART), the Gaussian process of regression (GPR), and the extreme gradient boosting tree (XGBoost). As ECC is a new material, the data available in the literature are not significant for ML, and thus, the Generative Adversarial Network (GAN) model is used for data augmentation. A virtual dataset of 1000 samples is successfully extracted by this model and is used for training while the original set is used for testing. The performance-based on this technique is phenomenal. Finally, the accuracy of all models is compared to find the best model.

## 2. Methodology

### 2.1. Machine Learning Models

This section elaborates on the machine learning models used in the research. Five models were used to predict four parameters, out of which three were related to Regression, and one was related to classification. Among the five models, Gaussian Process Regression (GPR) cannot be used for classification rest all can be used for all types of data. Broadly, these models are divided into three categories: (1) Artificial Neural Networks (ANN), (2) Regression Analysis, and (3) Regression Tree analysis.

#### 2.1.1. Artificial Neural Networks (ANN)

**Figure 1:**
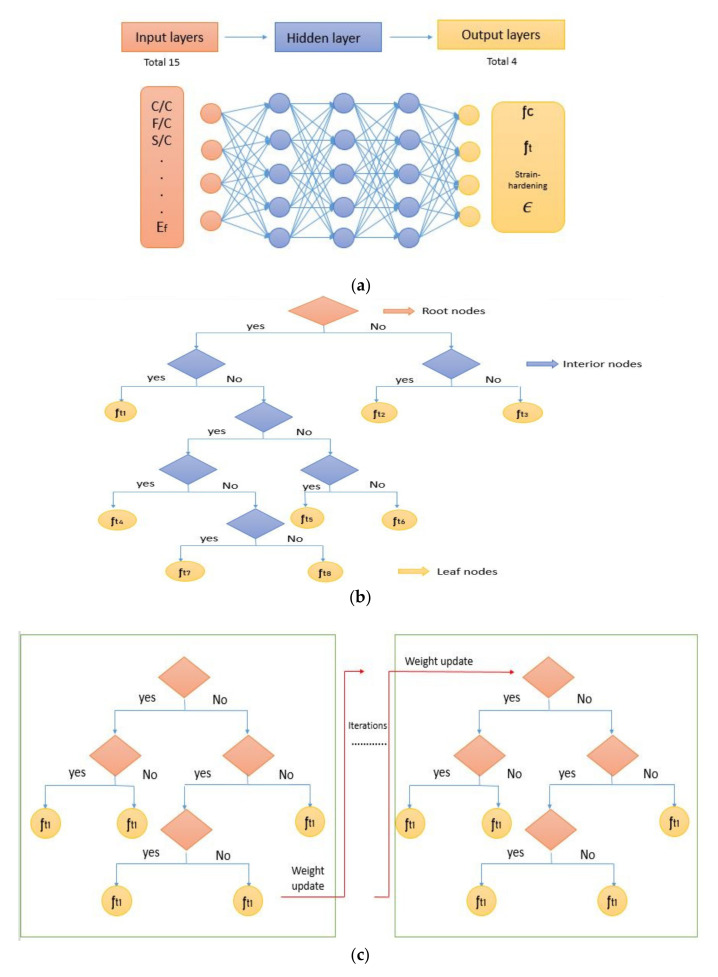
Machine Learning Models: (**a**) ANN; (**b**) CART; (**c**) XGBoost. f_t_ is the Tensile strength; C/C is the cement-to-cement Ratio; F/C is the Fly ash to cement ratio; S/C is the sand to cement ratio; E_f_ is the elastic modulus of fiber; f_c_ is the Compressive Strength; є is the tensile Strain Capacity.

ANN is a bio-inspired computational model that works the way human neurons work, which is why it has this name. This model contains three basic parameters (1) Input layer, (2) Output layer, and (3) Hidden layer. However, there are also some other parameters, but they were kept as default. For our problem, parameters for input and output are fixed. The hidden layer is a parameter that depends on data and defines the complexity of the model. The more hidden layers, the better the model can fit. However, it may cause overfitting, a situation in which data fits very well for the training dataset but performs poorly for the testing dataset [99]. Thus, the network was trained for different layers, and the optimum number of hidden layers was found by comparing the RMSE of training and validation datasets. Figure 1a depicts a typical ANN model.

#### 2.1.2. Regression Analysis

This is the most common family of models containing many different types with separate parameters, with the goal being to fit the data as closely as possible [100]. Common regression models include linear regression and polynomial regression.

#### 2.1.3. Regression Tree Analysis

Regression Tree is one of the most potent tools of ML for regression analysis. It performs the calculations in a hierarchal (Tree-like) manner. The number of trees is a parameter that defines its complexity; however, the model cannot be made too complex to avoid overfitting [101]. Common Regression Tree models include CART and XGBoost (iterative tree). Figure 1b,c shows typical CART and XGBoost model respectively.

### 2.2. Generative Adversarial Network (GAN)

**Figure 2:**
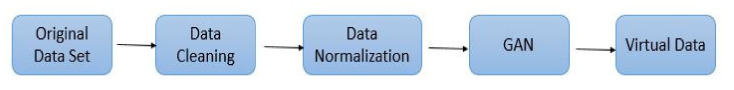
Processing of dataset.

In addition to the above models, a specialized data augmentation technique known as Generative Adversarial Network (GAN) was used. GAN is a technique used when the available data is not very large, allowing virtual data to increase accuracy by increasing the dataset [14]. Figure 2 show the processing of data to get virtual dataset.

### 2.3. Dataset

#### 2.3.1. Overview

For the development of the ML model, a dataset with 19 instances was used, from which 15 are input parameters, and 4 are outputs. Input parameters include matrix constituents and fiber properties as shown in Table 1. To cover a wide range of cement replacement materials, (1) matrix constituent: the cement-to-cement ratio, the fly ash-to-cement ratio, the sand-to-cement ratio, the coarse aggregate-to-cement ratio, the limestone powder-to-cement ratio, the slag-to-cement ratio, the silica fume-to-cement ratio, the metakaolin-to-cement ratio, the fiber content, the water-to-binder ratio, and the superplasticizer content was used. Major parameters that define (2) fiber properties are: the fiber length, the fiber diameter, the fiber tensile strength, and the fiber elastic modulus. 

Predicting the fracture response of the material is made possible using the dataset of conventional and HPFRC as well as the ECC samples. If only ECC samples were used to predict properties, there would have been a problem with the model of not differentiating the sample of other types of FRC. The model would have been treating input of any fiber concrete as an ECC and predicting higher values of strains considering strain-hardening. Training ML on the behavior of both FRC and ECC materials was made to overcome this issue. The trained model can predict the post cracking response based on variable differences in FRC and ECC.

The HPFRCCs sample data were included to capture the effect of coarse aggregate addition on the fracture attributes of the matrix. As ECC lacks coarse aggregate compared with FRC, the model might have confused the strain-softening behavior to the presence of coarse aggregates. Thus, HPFRCC data are added to avoid this mishap as it has coarse aggregate and shows stain-hardening simultaneously.

#### 2.3.2. Dataset Normalization

The data were collected from literature initially in raw form. Their range was drastically different, e.g., cement content was normally around 1, but other parameters such as fiber diameter or elastic modulus of fiber were in the range of hundreds. Therefore, data normalization was necessary so that the model could predict the sensitivity of each parameter, which ultimately affects the results. Therefore, to keep all the parameters between 0 and 1, the following normalization technique was used. For normalization, Equation (1) was used to keep the data between 0 and 1.

(1) $$x\;*=\frac{x-x\left(\min\right)}{x\left(\max\right)-x\left(\min\right)}$$

x is any original input parameter; x(min) is the minimum value of the similar parameter; x(max) is the maximum value of the parameter; x * is the normalized value of the parameter.

#### 2.3.3. Hyperparameter Tuning

Hyperparameter tuning is the most crucial parameter of machine learning models. In ANN, it corresponds to the number of hidden layers and learning rate, and in regression tree depends upon the number of branches. A simple iterative technique was used to find the performance of the model by changing the parameters. The best parameter for both training and validation sets was selected to counter underfitting and overfitting.

#### 2.3.4. Performance Evaluation

In order to test the performance accuracy of the model, three basic performance parameters were used for regression data to relate the predicted (Y_pre_) and actual results (Y_actual_) [102,103]. These three parameters include (1) root mean squared error (RMSE), (2) coefficient of determination (R^2^), and (3) Pearson correlation coefficient (R) as given by Equations (2)–(4).

(2) $$\mathrm{RMSE}\;=\;\sqrt{\frac{1}{n}.\sum_{i=1}^{n}{\left(\mathrm{Ypre}-\;\mathrm{Yactual}\right)}^{2}},$$

(3) $$R\;=\;\frac{\sum_{i}^{n}=1\left(\;Y\mathrm{pre}\;-\bar{\mathrm{Ypre}}\right).\left(\mathrm{Yactual}\;-\bar{\mathrm{Yactual}}\;\right)}{\sqrt{\sum_{i}^{n}=1{\left(\mathrm{Ypre}\;-\bar{\mathrm{Ypre}}\;\right)}^{2}}\;.\sqrt{\sum_{i}^{n}=1{\left(\;\mathrm{Yactual}-\bar{\mathrm{Yactual}}\;\right)}^{2}\;}}$$

(4) $$R^{2}\;=\;\frac{\sum_{i}^{n}=1\left(Y\mathrm{pre}-\bar{\;Y\mathrm{pre}}\right)}{\sum_{i}^{n}=1\left(\;\mathrm{Yactual}\;-\bar{\mathrm{Yactual}\;}\;\right)}$$

However, the classification data were evaluated based on fundamental parameters of AUC (Area under Curve), the area under the ROC curve, and accuracy in predicting the data. 

## 3. Implementations

### 3.1. Anomalous Data

Anomalous data are the outlier that can affect the model’s accuracy. Data are extracted from already published articles, including the hypothetical trails of different mixes. Fourteen such samples were removed, e.g., mix with 10% fibers, as it was an outlier, so it was removed. In the same way, reported compressive strength of over 200 MPa was also an outlier; therefore, it was also removed.

### 3.2. Hyperparameter Tuning

Table 2 shows the optimal hyperparameters for different machine learning models used for each output parameter. There are different sorts of interlinking between input and output parameters. Therefore, for better results, each hyperparameter was calculated using the simplified approach of using a loop and finding the optimal combination for which the error is minimal for both training and validation sets, along with keeping a special check on overfitting. Since the numbers of hyperparameters in some models were very high, tuning was done only on some of the hyperparameters, and the rest were taken as default. Table 2 shows Hyperparameters that were optimized. The hyperparameters missing in this table were kept as default. XGBoost showed good results using the default hyperparameters without any tuning.

### 3.3. Training Process

**Figure 3:**
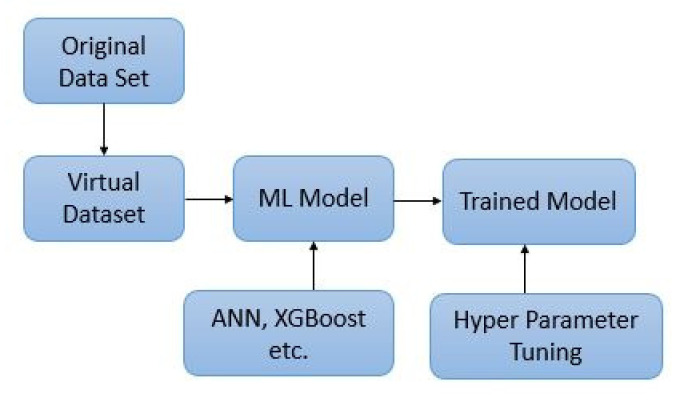
Training of model.

For the training process, all the optimal hyperparameters listed in Table 2 were used to train the machine learning model. Special attention was given to ensure the model neither be under-fitted nor overfit. The training process was done, and the performance of each model was calculated separately for the training and testing dataset as per parameters defined in Section 2.3.4. Figure 3 show the approach employed for training the model.

## 4. Results and Discussions

### 4.1. Predicted Results and Discussions

Based on above mentioned trained models, compressive strength, tensile strength, tensile strain, and post cracking behavior (whether strain hardening would occur or not) can be predicted. Table 3, Table 4 and Table 5 compare actual vs. predicted results of the defined output parameters. The prediction accuracy was measured in terms of R^2^ value and R-value. Its larger value corresponds to high prediction accuracy, while in the case of RMSE value, a low value indicates high accuracy. For post cracking behavior AUC and accuracy are used. Their higher value indicates higher prediction accuracy.

**Figure 4:**
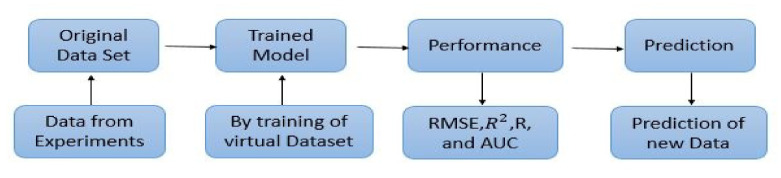
Working of predictive model.

The performance of each model is summarized in Table 6. The performance of the models was evaluated as per Section 2.3.4. The results of both testing and training data sets were compared to avoid under fitting and overfitting. Among all models, the XGBoost method shows the best accuracy for all the output parameters followed by ANN, GPR, CART, and SVM. This model gives RMSE for a training set of compressive strength, tensile strength, and ductility as 1.59, 0.2, and 0.163, respectively, while for the testing set RMSE is 2.35, 0.31, and 0.18, respectively, which was more accurate compared to the previous models [12] in which RMSE for a training set of compressive strength, tensile strength, and ductility as 2.5, 0.36, and 0.25, respectively, while for the testing set RMSE is 6.75, 0.774, and 0.785, respectively. The value of R^2^ for XGBoost was 0.99, 0.98, and 0.98 for compressive strength, tensile strength, and tensile strain, respectively, for training while 0.95, 0.95, and 0.97 for testing. XGBoost was able to classify the fracture behavior of the samples more accurately compared to other models. Its accuracy for classification was 98.5% and 98.4% for training and testing, respectively. The high accuracy of XGBoost is due to its iterative architecture, as shown in Figure 1c, which creates a better relationship between input and output parameters. Complete working process of predictive model is shown in Figure 4.

### 4.2. Validation of Predictive Models

**Figure 5:**
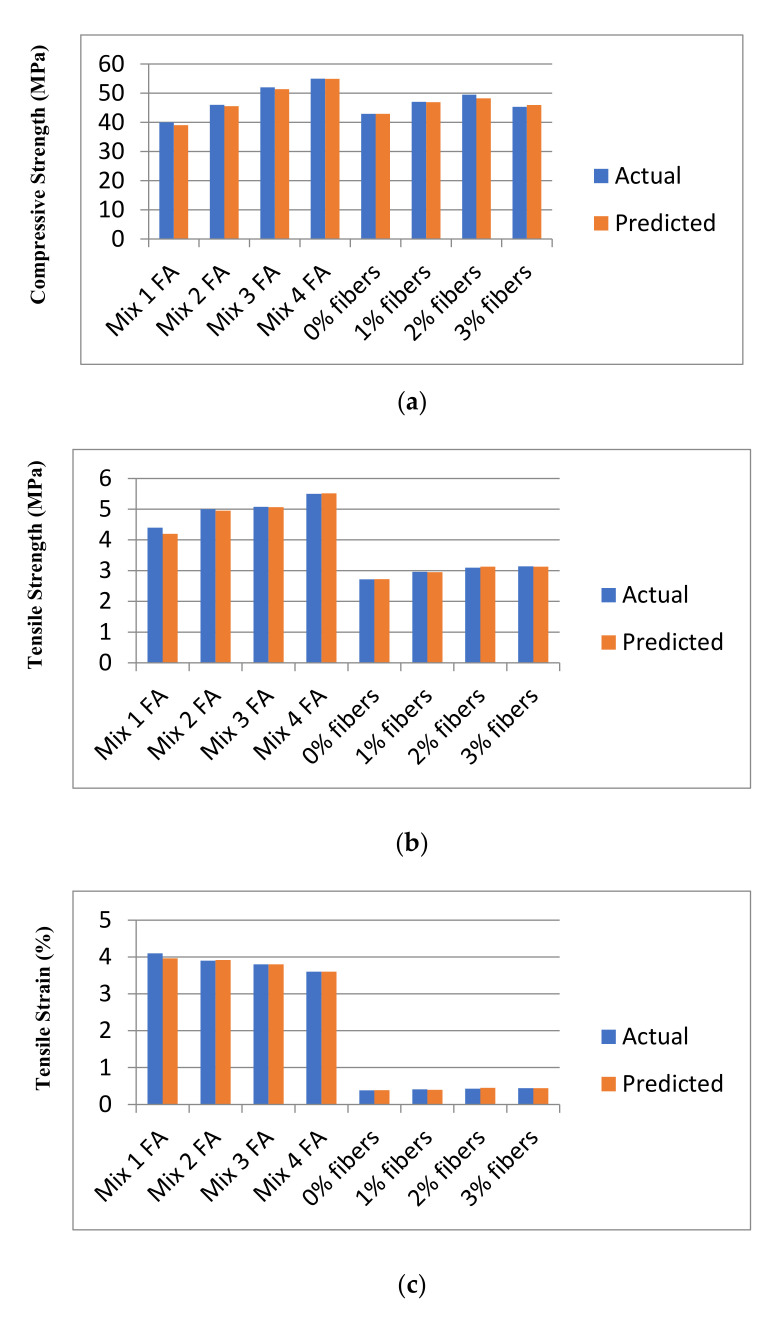
Comparison of prediction using XGBoost against the test results (**a**): Compressive Strength; (**b**) Tensile Strength; (**c**) Tensile strain.

It is clear from Table 6 that the XGBoost model has the maximum accuracy compared to the other models. Thus, it was used to predict compressive strength, tensile strength, tensile strain, and the post-cracking response of samples that are not included in any dataset (neither in original nor in virtual). The model was practiced for validation by published experiments’ data. Samples with one varying parameter were checked. Two types of samples with varying percentage content of flyash and fiber content were tested. Figure 5 shows the comparison of actual v/s predicted properties of samples. Results reveal that XGBoost is accurate in predicted values, and classification made by the model about post cracking behavior is also 100% true.

## 5. Conclusions and Recommendations

This research was able to develop a new way to predict fracture properties (i.e., Mechanical properties, ductility, and the post-cracking response) of FRC, using the aid of machine learning. Five models were developed to predict four outputs with 15 input parameters of FRC. The performance of each model was evaluated, and from those following conclusions were made:

- The predictive models are accurate enough to replace the extensive experimentation trails required for optimizing FRC according to desired needs.
- These models can be effectively used to bifurcate fracture behavior as strain hardening or softening based on selected inputs as they are well trained for both types of behavior. XGBoost model shows 98.4% accuracy in segregating the fracture response of fiber-reinforced matrices.
- The above-proposed models can realistically be used to predict mechanical properties, ductility, and post-cracking behavior of both the traditional and high-performance FRC. Among all models, the XGBoost model shows the best accuracy for all the output parameters. This model gives RMSE for a training set of compressive strength, tensile strength, and ductility as 1.59, 0.2, and 0.163, respectively, while for the testing set RMSE is 2.35, 0.31, and 0.18, respectively. These performance indicators of RMSE were more accurate than previously implemented models [12].
- GAN was used to successfully produce a virtual dataset of 1000 samples using the original dataset. This virtual dataset further increased the accuracy of the models.
- These models can also be optimized in a way to make the mix economic with improved mechanical properties along with minimizing the environmental impacts (e.g., carbon footprint and reuse of waste products)

Future research is needed to find out other parameters and their dependence on different important parameters of FRC, e.g., fresh properties, durability properties, use of other types of cement, or incorporating the packing density concept for high strength concrete. More research is also needed using these models for other types of special-purpose concretes.

## Figures and Tables

**Table 1:** Input parameters details.

No:	Input Variable	Range	Unit	Mean	Standard Deviation
1	Cement-to-cement ratio	1	1	1	0
2	Fly ash-to-cement ratio	0–4.4	1	0.64	0.97
3	Sand-to-cement ratio	0–6.5	1	1.3	0.9
4	Coarse aggregate-to-cement ratio	0–7.24	1	1.15	1.53
5	Limestone powder-to-cement ratio	0–6.5	1	0.052	0.39
6	Slag-to-cement ratio	0–4	1	0.109	0.38
7	Silica fume-to-cement ratio	0–0.375	1	0.037	0.08
8	Metakaolin-to-cement ratio	0–0.5	1	0.011	0.05
9	Fiber content	0–7	%	1.59	0.89
10	Water-to-binder ratio	0.14–0.99	1	0.42	0.16
11	Superplasticizer content.	0–6	%	1.1	1.5
12	Fiber length	0–100	mm	16.7	12
13	Fiber diameter	0–1000	µm	176	259
14	Fiber tensile strength	0–4475	MPa	1476	800
15	Fiber elastic modulus	0–228	GPa	80.2	71

**Table 2:** The optimal hyperparameters for ML models.

Method	Hyperparameters	Range	Optimal Value for Different Parameters			
			Compressive Strength	Tensile Strength	Strain-Hardening	Tensile Strain Capacity
ANN	Hidden layer size	1–100	55	64	65	68
SVM	C	1–20	10	6	12	9
	Gamma	0.1–1	0.4	0.3	0.7	0.6
	Epsilon	0.1–2	0.1	0.1	0.1	0.1
CART	Maximum depth	2–10	2	5	2	4
	Minimum samples leaf	2–10	2	2	2	2
GPR	Kernel Scale	0.001–1	0.024	0.034	0.028	0.022
	Sigma	0.0001–254	0.0054	0.06	0.00326	0.00125

**Table 3:** Comparison of predicted and actual values of mechanical properties.

Compressive Strength	Tensile Strength
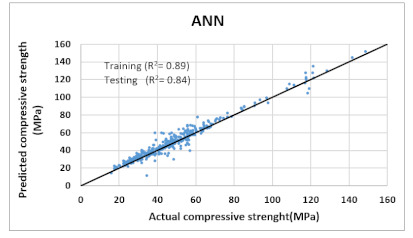	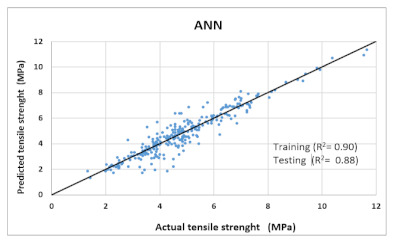
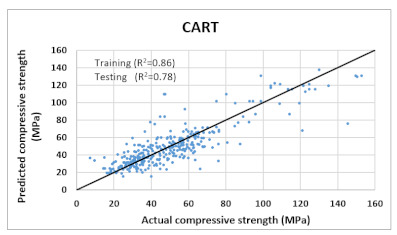	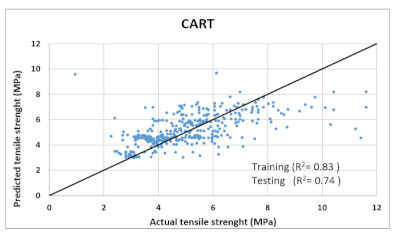
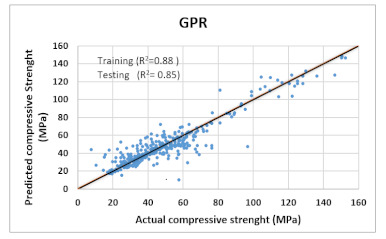	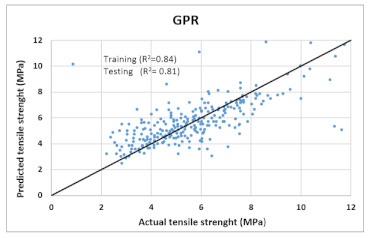
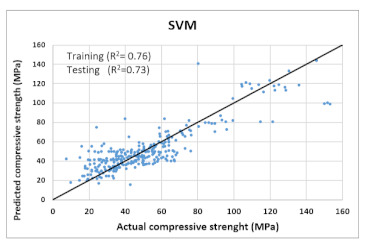	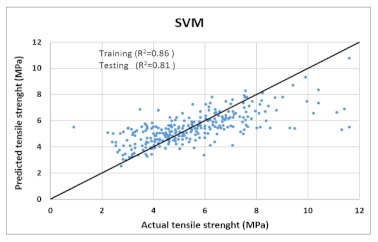
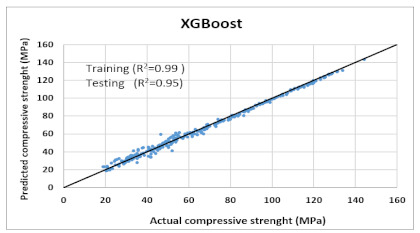	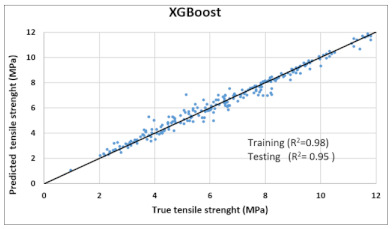

**Table 4:** Comparison of predicted and actual values of ductility properties.

Tensile Strain	
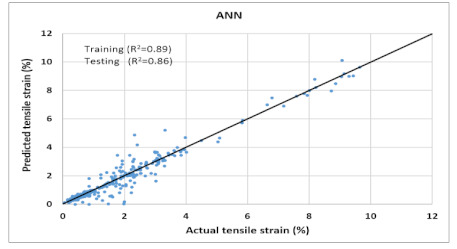	
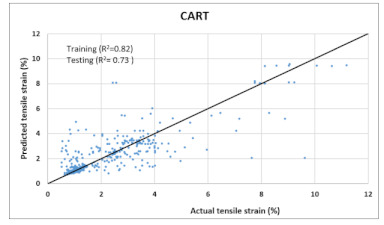	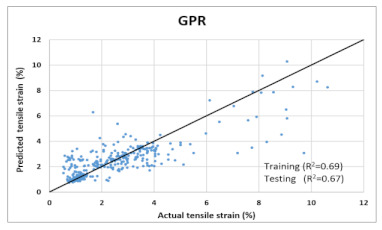
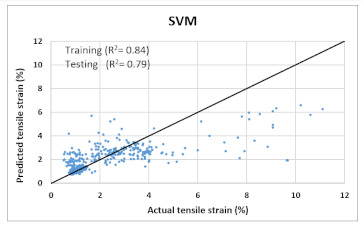	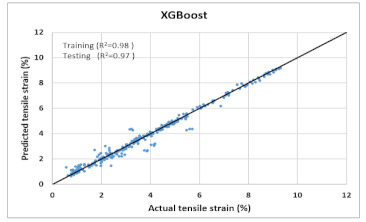

**Table 5:** AUC and Confusion matrix for predicting Post-cracking response.

Strain-Hardening	
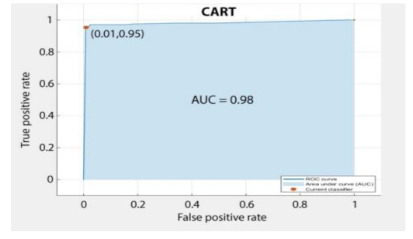	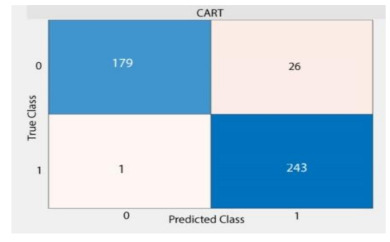
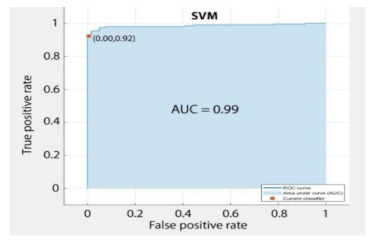	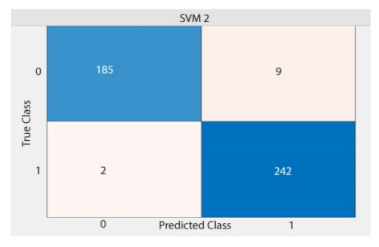

**Table 6:** Evaluation of predicted results.

Model	Set	Evaluation	Compressive Strength	Tensile Strength	Strain-Hardening	Tensile Strain Capacity
ANN	Training	RMSE	5.7	0.7549	-	0.7189
		R^2^	0.893	0.902	-	0.899
		R	0.94	0.941	-	0.93
		AUC	-	-	0.98	-
		Accuracy	-	-	96.3%	-
	Testing	RMSE	7.3	1.038	-	0.7726
		R^2^	0.84	0.885	-	0.86
		R	0.92	0.937	-	0.91
		AUC	-	-	0.97	-
		Accuracy	-	-	94%	-
SVM	Training	RMSE	12.05	1.4	-	1.3
		R^2^	0.76	0.86	-	0.84
		R	0.88	0.927	-	0.9
		AUC	-	-	0.98	-
		Accuracy	-	-	97.5%	-
	Testing	RMSE	12.49	1.8	-	1.44
		R^2^	0.73	0.81	-	0.79
		R	0.84	0.9	-	0.87
		AUC	-	-	0.96	-
		Accuracy	-	-	97%	-
CART	Training	RMSE	6.025	1.8	-	1.1
		R^2^	0.868	0.83	-	0.82
		R	0.91	0.9	-	0.89
		AUC	-	-	0.98	-
		Accuracy	-	-	94.67%	-
	Testing	RMSE	11.97	1.97	-	1.357
		R^2^	0.78	0.74	-	0.73
		R	0.87	0.855	-	0.85
		AUC	-	-	0.95	-
		Accuracy	-	-	93%	-
XGBoost	Training	RMSE	1.59	0.2	-	0.163
		R^2^	0.99	0.983	-	0.98
		R	0.995	0.9918	-	0.9927
		AUC	-	-	0.998	-
		Accuracy	-	-	98.5%	-
	Testing	RMSE	2.35	0.31	-	0.18
		R^2^	0.95	0.95	-	0.974
		R	0.97	0.97	-	0.991
		AUC	-	-	0.99	-
		Accuracy	-	-	98.4%	-
GPR	Training	RMSE	8.6	1.1	-	1.007
		R^2^	0.88	0.84	-	0.69
		R	0.92	0.89	-	0.81
	Testing	RMSE	9.1	1.37	-	1.16
		R^2^	0.85	0.81	-	0.67
		R	0.89	0.86	-	0.8

## Data Availability

Data is contained within the article.

## Abbreviations

FRC	Fiber Reinforced Concrete
ECC	Engineered Cementitious Concrete
HPFRCCs	High Performance Fiber Reinforced Cementitious Composites
ANN	Artificial Neural Network
SVM	Support Vector Machine
GPR	Gaussian Process of Regression
XGBoost	Extreme Gradient Boosting Tree
CART	Classification and Regression Tree
GAN	Generative Adversarial Network
ML	Machine Learning
RMSE	Root Mean Squared Error
AUC	Area under Curve
R^2^	Coefficient of determination
R	Pearson correlation coefficient
